# Not All Antibodies Are Created Equal: Factors That Influence Antibody Mediated Rejection

**DOI:** 10.1155/2017/7903471

**Published:** 2017-03-08

**Authors:** Carrie L. Butler, Nicole M. Valenzuela, Kimberly A. Thomas, Elaine F. Reed

**Affiliations:** Department of Pathology and Laboratory Medicine, David Geffen School of Medicine, University of California, Los Angeles, CA, USA

## Abstract

Consistent with Dr. Paul Terasaki's “humoral theory of rejection” numerous studies have shown that HLA antibodies can cause acute and chronic antibody mediated rejection (AMR) and decreased graft survival. New evidence also supports a role for antibodies to non-HLA antigens in AMR and allograft injury. Despite the remarkable efforts by leaders in the field who pioneered single antigen bead technology for detection of donor specific antibodies, a considerable amount of work is still needed to better define the antibody attributes that are associated with AMR pathology. This review highlights what is currently known about the clinical context of pre and posttransplant antibodies, antibody characteristics that influence AMR, and the paths after donor specific antibody production (no rejection, subclinical rejection, and clinical dysfunction with AMR).

## 1. Introduction

Antibody mediated rejection (AMR) is a major contributor to rejection risk and allograft loss in solid organ transplantation [1, 2]. AMR diagnostic criteria were first established in cardiac [3] and renal [4] transplantation and have recently been described for pancreas [5] and lung transplantation [6] and, although historically controversial, are proposed for liver [7] and intestinal [8] allografts as well. AMR incidence is approximately 10–20% in cardiac [9], 5–8% in renal [10], 4–25% in lung [11, 12], and 24% in liver [13] transplant. Central features of AMR pathology include endothelial cell (EC) swelling, microvascular inflammation, and intravascular CD68+ macrophages with or without complement deposition. Antibodies, most notably those specific for human leukocyte antigen (HLA), mediate effector functions that manifest in the histopathology of AMR. HLA are the most polymorphic genes in the human genome and as such result in the development of alloantibodies when an exact match is not found, as the donor allograft contains foreign HLA. The presence of HLA donor specific antibodies (DSA) is highly indicative of AMR [14, 15]. The advent of HLA DSA detection methods [16–18] has led to studies identifying the correlation of HLA DSA with more severe AMR, yet we still are unable to fully predict how harmful or “pathogenic” DSA will be. Further complicating matters is the recent association of non-HLA antibodies with allograft rejection. A greater understanding of all the factors (donor and recipient characteristics and antibody attributes) that contribute to rejection is needed to enhance the predictive performance of risk assessments and better determine which patients are at an increased risk for AMR. This review will address the clinical context of pre- and posttransplant antibodies, HLA and non-HLA antibody characteristics that influence AMR, and the three outcomes (stable function, subclinical dysfunction, and clinical dysfunction with AMR) mediated by these antibodies.

## 2. HLA Antibodies

### 2.1. Pretransplant

Pretransplant sensitization can occur from prior transplants, blood transfusions, pregnancy, and mechanical assist devices (in heart failure) resulting in autoantibody formation. Allosensitization affects approximately 6–9% of cardiac transplant candidates [19, 20] and 23% of renal transplant candidates prior to transplantation [21]. Patients who are presensitized have a significantly increased risk of developing AMR within the first three years after cardiac transplantation compared to those who are not sensitized [22]. Preformed HLA antibodies were also associated with AMR in kidney transplantation [23–25]. In a multicenter prospective clinical study, kidney allograft recipients with HLA antibodies were associated with an increased risk for graft failure 1 year after transplant [26]. In liver transplantation, preformed DSA has been associated with an increased risk of AMR [7, 27]. Roux et al. found that preformed HLA DSA was associated with AMR, chronic dysfunction, and graft loss in a lung transplant cohort with 2-year follow-up [28].

### 2.2. Posttransplant

After transplantation, 24% of renal allograft recipients will develop de novo HLA DSA within ten years [29] and approximately 25% of cardiac allograft recipients will develop de novo HLA DSA within thirteen years [30]. Nearly one-third of low risk patients (first transplant, no DSA) develop de novo DSA by 12 years after transplant [31]. De novo DSA development rates are 25–50% after lung transplantation [6]. Additionally, long-term posttransplant follow-up of renal allograft recipients revealed a significant decline in the ten-year graft survival rate for recipients that developed de novo antibodies to HLA [32] compared to those that did not. Smith et al. reported that de novo and persistent DSA postcardiac transplant were associated with poor long-term patient survival [30]. De novo DSA in liver transplantation is also associated with AMR [27, 33]. Many studies in lung transplant do not address the temporal timing of DSA potentially because AMR has only recently been recognized in lung transplantation and the presence of circulating DSA (regardless of preformed or de novo) is a key diagnostic standard [6]. However, multiple studies have found an associated risk of AMR in patients with DSA in lung transplantation [12, 34].

## 3. Non-HLA Antibodies

### 3.1. Pretransplant

Non-HLA antigens have been shown to be expressed intracellularly, on the EC cell surface and to apoptotic cells [35]. Non-HLA antibodies can occur independently or can occur concurrently with HLA DSA within patients, sometimes creating a synergistic effect on the allograft [36]. Additionally, antibodies specific for angiotensin II type 1 receptor (AT_1_R) can precede de novo HLA DSA [36]. AT_1_R antibodies are autoantibodies. Pretransplant AT_1_R antibodies have been associated with AMR in kidney [37, 38] and heart [39] transplant recipients. MICA is a polymorphic nonclassical class I antigen that is closely linked to the HLA-B locus and is upregulated on endothelial and epithelial cells during cellular stress. Antibodies to MICA have been found in the serum of renal [40] transplant recipients and were associated with humoral rejection and graft loss. Perlecan is a heparin sulfate proteoglycan that is cleaved by cathepsin-L to form a C-terminal fragment called LG3, as it contains three laminin-like globular domains. Pretransplant LG3 antibodies have been found to be associated with acute vascular rejection independent of HLA DSA in kidney transplant recipients [41]. Patients with preformed collagen V and K*α*-tubulin antibodies were at an increased risk of developing HLA DSA, and bronchiolitis obliterans syndrome (BOS) [42], a manifestation of chronic rejection.

### 3.2. Posttransplant

AT_1_R antibodies are associated with AMR in cardiac [39] and renal [43–45] transplantation. Others have reported that concomitant HLA and AT_1_R antibodies in renal and cardiac transplantation increased the risk of AMR and decreased graft survival [36, 46]. Endothelin type A receptor (ET_A_R) antibodies have also been reported in renal [47] and cardiac [39] transplantation. Antibodies against AT_1_R and ET_A_R have also been shown to be increased in lung allograft recipients with cystic fibrosis [48]. Antibodies to MICA have been found in the serum of renal [40] and cardiac [49] transplant recipients, although conflicting evidence exists about the independent pathogenic role of MICA in chronic rejection [50, 51]. Posttransplant LG3 antibodies have been found in renal transplant recipients [41, 52]. Collagen V (Col V) is an extracellular matrix protein expressed on the lung interstitium and lung epithelial cells. Col V antibodies have been found in sera from lung allograft recipients diagnosed with bronchiolitis obliterans syndrome (BOS), a manifestation of chronic rejection [53, 54]. Additionally, Col V autoantibodies are associated with AMR and cardiac allograft vasculopathy (CAV) in cardiac transplant recipients [55] and transplant glomerulopathy in renal allograft recipients [56].

## 4. Three Paths after DSA

**Figure 1:**
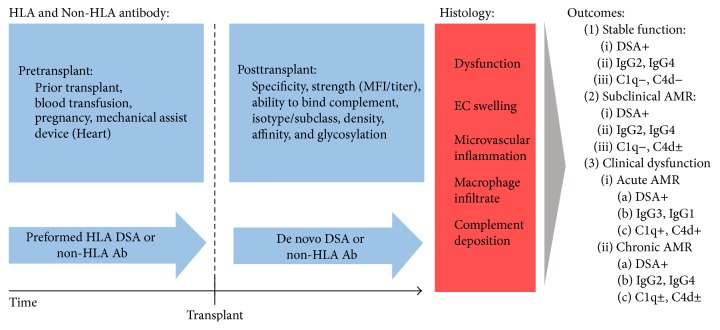
Factors influencing AMR. Schematic of the antibody components that influence AMR's pathogenesis. Depicted are the antibody factors (blue) that influence AMR pathology (shown in red). Antibody factors influencing AMR include sensitization pretransplant and antibody attributes such as specificity, ability to bind complement, isotype/subclass, strength (MFI/titer), density, affinity, and glycosylation. AMR histology (red) includes graft dysfunction, endothelial cell (EC) swelling, microvascular inflammation, and macrophage infiltrate and can occur with or without complement deposition. The three outcomes after DSA include stable function, subclinical AMR and clinical dysfunction with AMR (either acute or chronic). Stable function in the presence of DSA is typically seen in those patients with IgG2/IgG4 antibodies that do not show signs of complement binding antibodies (C1q−, C4d−). Subclinical AMR is typically seen in those patients with IgG2/IgG4 antibodies that may show signs of complement binding antibodies (C1q−, C4d±). Clinical dysfunction with AMR can be grouped into acute or chronic AMR. Acute AMR is typically seen in those patients with IgG3/IgG1 antibodies that are complement binding antibodies (C1q+, C4d+). Chronic AMR is typically seen in those patients with IgG2/IgG4 antibodies that may or may not include complement binding antibodies (C1q±, C4d±).

Patients with DSA do not represent a uniform category. Patients are either transplanted with no allosensitization, with HLA antibodies but no donor specific antibodies (3rd party), or with preformed DSA. Additionally, patients with a history of sensitization may never have circulating DSA detected in screening protocols, even though they have formed T and B cell alloimmune memory. Despite strong evidence that DSA are associated with increased rejection incidence and reduced graft survival, it is unknown why a subset of patients with DSA does* not* experience poorer graft outcomes in these studies [31, 57, 58]. This creates uncertainty about how to manage patients who exhibit DSA on routine monitoring but have no clinical signs of graft dysfunction or whether a preformed HLA DSA of a certain strength or titer can be safely crossed. Extraordinarily high levels of DSA (>10,000 MFI in our experience), especially to HLA class I antigens, have been shown to be cytotoxic and place patients at risk of hyperacute rejection via complement activation; such strong DSA are typically avoided with the exception of liver transplantation [59, 60]. Transplant recipients with DSA can exhibit overt rejection (acute or chronic) with clinical dysfunction, indolent dysfunction (slow decline in graft function) with subclinical rejection on protocol biopsy, or stable function and normal biopsy (Figure 1).

### 4.1. Clinical Dysfunction with AMR

Evidence of clinical allograft dysfunction is an important consideration in diagnosis of symptomatic (clinical) AMR. Nearly half of patients transplanted with preformed DSA experienced AMR, compared with less than 1% in those without pretransplant DSA [61]. Of renal transplant recipients with preformed DSA who developed AMR, the majority were flow crossmatch positive [61–63]. De novo DSA is often observed at the same time as clinical dysfunction [32], and the vast majority of patients presenting with allograft functional impairment and dnDSA were nonadherent [32]. Thus patients are more likely to develop AMR if their DSA is strong enough to cause a positive flow crossmatch and more likely to experience graft dysfunction if they were medication nonadherent. In the long-term, patients experiencing clinical dysfunction with AMR have the worst 5-year graft survival compared with TCMR or no rejection [58].

### 4.2. Subclinical AMR

Studies evaluating protocol biopsies have reported a high incidence of subclinical AMR that is likely missed by monitoring strategies that biopsy only for cause. One-year surveillance biopsies in DSA+ patients with stable function nonetheless often revealed C4d deposition and peritubular capillaritis [32], indicative of “smoldering” inflammation not present in patients without DSA. Similarly, Loupy et al. showed that 14% of clinically stable renal transplant recipients had evidence of subclinical AMR on one-year surveillance biopsy [57]. The majority of these patients had performed DSA. Importantly, these studies have demonstrated that patients with subclinical AMR (i.e., no acute dysfunction) fare significantly worse than their DSA negative counterparts [57], with faster decline in GFR of renal allografts [31, 57, 61] and higher rates of CAV in cardiac allografts [64–66]. Renal transplant recipients with subclinical AMR who received treatment with plasmapheresis unfortunately had comparable outcomes to those who were not untreated, and both had a significant decrement in 5-year survival compared with AMR-free controls [61]. Similarly, half of cardiac transplants that failed more than one year after transplant due to chronic rejection had a history of subclinical AMR [67]. While patients with clinically symptomatic AMR fare worse than those with subclinical AMR, both groups have significantly reduced 10-year outcomes compared with stable, DSA negative patients [31].

### 4.3. DSA with Stable Function and No Rejection

Intriguingly, up to half of patients with preformed DSA did* not* have rejection, subclinical AMR, or otherwise, at the time of one-year biopsy [57, 68]. Approximately 20% of stable patients with no evidence of rejection on protocol biopsy also had DSA. Thus, a critical, yet unanswered, question is which patient, donor, and antibody characteristics might protect from rejection and graft dysfunction in the presence of DSA, a question which is addressed in part in the next section.

## 5. Mechanisms of Antibody Mediated Graft Injury

### 5.1. HLA Antibodies

Antibodies mediate allograft injury and contribute to graft pathology through three main types of effector functions: EC activation, complement activation, and leukocyte interaction/activation. Alterations in these effector functions modulate rejection severity. Antibody characteristics, such as titer, isotype/subclass, glycosylation, and affinity, can influence these effector functions. The interface between the allograft and its recipient is the thin layer of donor EC lining the walls of the blood vessels supplying nutrients to the allograft. Gene profiling studies of renal [69–71] and cardiac biopsies [72] undergoing AMR identified EC activation as a significant contributor to graft pathology. Crosslinking of HLA expressed on the surface of EC by DSA triggers a series of intracellular signaling events and activation of immune responses, which are manifested in the histopathological findings in AMR pathology [73]. DSA binding to HLA induces EC activation, resulting in P-selectin expression and mammalian target of rapamycin (mTOR) dependent cellular migration, proliferation, and protein synthesis [74–77]. Positive staining of phosphorylated mTOR signaling proteins including S6 kinase and S6 ribosomal protein in the capillary EC of endomyocardial biopsies strongly correlated with diagnosis of AMR [78, 79]. EC activation facilitates chemokine expression leading to leukocyte recruitment to inflammatory sites [80]. In addition, increased EC and smooth muscle proliferation results in a thickening of the tunica intima [81], a hallmark of chronic AMR in all solid organ transplant patients [3]. Antibody titer affects EC signal transduction and subsequent EC activation, as increasing quantities of HLA antibody result in augmented FGFR expression and cellular proliferation [82], whereas decreased antibody titer results in upregulation of prosurvival genes and antiapoptotic proteins in EC [83]. An additional antibody-independent factor that influences HLA-mediated signaling is the density of HLA molecule expression on the EC surface. HLA antigen expression on graft endothelium is increased during allograft rejection in response to IFN*γ* and induces CIITA activation and subsequent HLA Class II expression [84–87]. The density of HLA on the surface of EC directly affects the degree of DSA binding to the graft and downstream effector functions.

Although complement deposition is no longer necessary for AMR diagnosis, complement binding DSA increases a patient's risk for kidney allograft loss five years after transplant [88] and complement binding antibodies were more predictive, than HLA DSA alone, of an increased risk for AMR and decreased graft survival in cardiac transplant ten years after transplant [89]. Antibody isotype and subclass play a significant role in induction of the classical complement pathway, with IgM, IgG3, and IgG1 having the highest degree of complement activation [90]. Antibody affinity mediated by IgG hexamers has been shown to be more efficient than isolated IgG molecules at activating the complement cascade [91]. Complement binding is also increased when there is an increase in the amount of antibody bound to cells [92, 93]. High panel reactive antibodies (PRA) are associated with increased complement activation [92]. Lastly, polymorphisms within the complement genetic locus could potentially affect the degree of complement activation [94, 95], whereas differential expression of complement regulatory proteins by the donor tissue could also affect the response of endothelium to complement components [96]. With respect to the downstream effects of complement activation, Jane-wit et al. demonstrated that complement activation and deposition on EC resulted in noncanonical NF*κ*B activation [97] whereas Cravedi et al. highlighted a role for complement activation in promotion of a Th1 response during alloimmune reactions [98]. Taken together, this information highlights the potential contributions of DSA on complement activation and promotion of alloimmunity.

Leukocyte recruitment and activation are a common histological feature of AMR. Macrophage infiltration is observed in heart [3] and renal [99] AMR and predicts a worse outcome [100]. Neutrophil recruitment is seen in lung transplantation and intragraft natural killer (NK) cells have been identified by molecular microscopy techniques in renal [71] and cardiac biopsies [72] diagnosed with AMR. IgG subclass dictates Fc receptor binding affinity [101, 102], thereby influencing leukocyte recruitment. Several studies have attempted to characterize the repertoire of DSA immunoglobulin subclasses in transplant recipients and correlate them with allograft outcomes. Their results have suggested that IgG3 DSA are a driver of acute AMR [103, 104], while IgG4 correlates more closely with subclinical AMR [105] and chronic rejection [106, 107]. Moreover, different terminal moieties in the Fc glycan of IgG have been demonstrated to change the inflammatory nature of antibodies. Sialylated IgG promotes a more tolerant environment, whereas glycans with terminal galactose residues are affiliated with a proinflammatory response [108]. Altered P-selectin expression allows for an increase in leukocyte recruitment [77 depending on subclass, by engaging Fc*γ*Rs, 109], a common histological feature across solid organ transplant [3, 6, 109]. DSA also facilitated NK cell-mediated antibody-dependent cellular cytotoxicity (ADCC) in an IFN*γ* and cell-contact dependent manner [110]. Collectively, the effector functions of DSA, while multifactorial themselves, are even more complex and multilayered when antibody characteristics are altered.

### 5.2. Non-HLA Antibodies

There is less mechanistic data for non-HLA antibodies in the pathogenesis of AMR. However, non-HLA antibodies can also mediate EC activation and complement activation and leukocyte interaction/activation. Non-HLA antibodies that activate EC can increase the expression of HLA class I and II and have been shown to develop independently or in conjunction with HLA DSA [36, 111]. AT_1_R antibodies mediate endothelial cell activation and vasoconstriction by binding to the second extracellular loop of the AT_1_R protein and act as an angiotensin II agonist promoting downstream activation of AP-1 and NF-*κ*B [112]. AT_1_R and ET_A_R antibodies frequently occur together in patient sera [111]; but there are no studies linking their pathologic mechanism. DSA bound to EC are also capable of activating the classical complement pathway, resulting in detection of C4d deposition along the capillary walls within allograft biopsies [113–115]. C4d deposition in graft histology has only been detected in a subset of patients with AT_1_R antibodies suggesting that the mechanism of injury for AT_1_R antibodies is not the complement pathway [43, 44, 112]. However, other non-HLA antibodies such as MICA can activate complement [116]. Evidence suggests that Col V antibodies increase IL-17 and IFN*γ* secreting T cells [117]. LG3 antibodies promote the migration of smooth muscle cells or mesenchymal stem cells to cause vascular injury [118].

Experimental models and clinical experience demonstrate that anti-donor HLA and non-HLA antibodies exhibit pathogenic functions through multiple mechanisms that likely have extensive crosstalk. AMR manifests as a broad spectrum both histologically and symptomatically. Across solid organs, the microvasculature is the principal target of antibody mediated injury. A single uniform approach to prevent graft injury and loss in the setting of donor specific antibodies will probably not be effective for all patients, and personalized therapies tailored to address unique patient and donor features will be needed to protect from AMR and chronic rejection. Non-HLA antibodies have also been associated with TCMR in renal transplantation [119] suggesting additional mechanisms of action that promote distinct graft pathology phenotypes compared to HLA DSA. While these histopathological features are diagnostically useful, they are an in situ read-out of the downstream effects of DSA-mediated effector functions. Recent work is uncovering additional mechanisms by which DSA can mediate immune activation. Further studies are needed to delineate the crosstalk between HLA and non-HLA antibodies and their synergistic effect on graft injury and to assess their incidence across different organ types.

## 6. Conclusions and Future Directions

Collectively, data on antibody pathogenicity defined by the antibody specificity, isotype, and ability to activate EC and complement can lead to different effector functions that mediate different pathological outcomes. Further studies to clarify which HLA and non-HLA antibody attributes (strength, subclasses, glycosylation, and affinity) contribute to subclinical, acute, and chronic AMR would be useful in order to identify biomarkers of different outcomes. Employment of newer techniques, such as the “molecular microscope,” can provide additional insight into the active transcriptome in the graft tissue, allowing a measurement of the local inflammation and the transcriptome signature for AMR [68]. Clinical research to determine how effective these parameters are at risk stratifying patients is needed. Enhanced understanding of the HLA and non-HLA mechanisms in allograft injury is needed to help identify additional therapeutic targets and further understand the potential synergistic relationship between them. Allograft rejection can occur throughout the lifetime of a transplanted organ and as such further understanding of the sensitization and pathologic mechanisms is needed to better risk stratify patients and achieve the goal of increasing long-term survival.
